# Hemophagocytic Syndrome: A Serious Complication in Non-Hodgkin Lymphoma

**DOI:** 10.7759/cureus.81705

**Published:** 2025-04-04

**Authors:** Samantha González Delgado, Rebeca Deyanira González García, Luis Paulo Ugarte Peláez, Diana Laura Morales Wong, Itzel Araceli Ortiz Meza

**Affiliations:** 1 Internal Medicine, Universidad Autónoma de Nuevo León, San Nicolás de los Garza, MEX; 2 Internal Medicine, Hospital Universitario de Monterrey, San Nicolás de los Garza, MEX; 3 Hematology, Hospital Universitario de Monterrey, San Nicolás de los Garza, MEX; 4 Pathology, Hospital Universitario de Monterrey, San Nicolás de los Garza, MEX

**Keywords:** cerebral metastasis, hemophagocytic lymphohistiocytosis (hlh), large b cell lymphoma, mental status changes, severity of disease

## Abstract

Hemophagocytic syndrome (HPS) is a common complication in pediatric populations but a rare disease in adults. Also known as hemophagocytic lymphohistiocytosis (HLH), the few cases reported in adults have been primarily caused by severe infections and hematologic malignancies. We highlight the importance of considering HPS as a differential diagnosis in patients with persistent inflammatory conditions. This report describes the aggressive clinical course of a 58-year-old female patient. Prompt recognition of HPS may improve outcomes in such cases.

## Introduction

Hemophagocytic syndrome (HPS), also known as hemophagocytic lymphohistiocytosis (HLH), is a hyperinflammatory syndrome characterized by dysregulated activation of macrophages and cytotoxic lymphocytes, leading to cytokine storm and multiorgan damage [1, 2]. Hemophagocytic syndrome is classified as primary (genetic) or secondary (acquired). Primary HPS is associated with mutations in genes such as perforin-1, whereas secondary HPS is more common and linked to infections, autoimmune disorders, or malignancies [3]. In secondary HPS, a cytokine release is the main trigger leading to a hyperinflammatory condition; according to malignant-associated HPS, lymphoma represents the most common trigger, exhibiting poorer outcomes [4]. Clinical features include persistent fever and cytopenia; elevated ferritin levels often exceeding 1000 ng/mL (normal range: 11-307 mcg/L), aiding diagnosis. Hematologic neoplasms are frequent triggers and correlate with poor prognosis [2, 5].

## Case presentation

We present the case of a 58-year-old female with type 2 diabetes and hypertension. The patient reported four days of general discomfort and diminished appetite, along with six days of melenic stool.

She consulted a private physician who ordered blood samples and an endoscopy. Laboratory findings revealed severe anemia with hemoglobin (Hb) at 6.8 g/dL (normal range: 11.6-15 g/dL). The endoscopy identified a gastric fundus ulcer and hemorrhagic gastropathy. Upon arrival at the emergency department, the patient had severe abdominal pain (10/10) and hypotension, and laboratory findings showed moderate anemia with Hb at 8.43 g/dL (normal range: 11.6-15 g/dL) and hematocrit at 26.8% (normal range: 30%-48%). There were no abnormalities in coagulation tests: prothrombin time 12.5 seconds (normal range: 10-15 seconds), partial thromboplastin time 26.8 seconds (normal range: 25-35 seconds), international normalized ratio 1.13 (normal range: 0.8-1.2). Notably, lactate dehydrogenase (LDH) was elevated to 593 U/L (normal range: 125-220 U/L).

**Figure 1:**
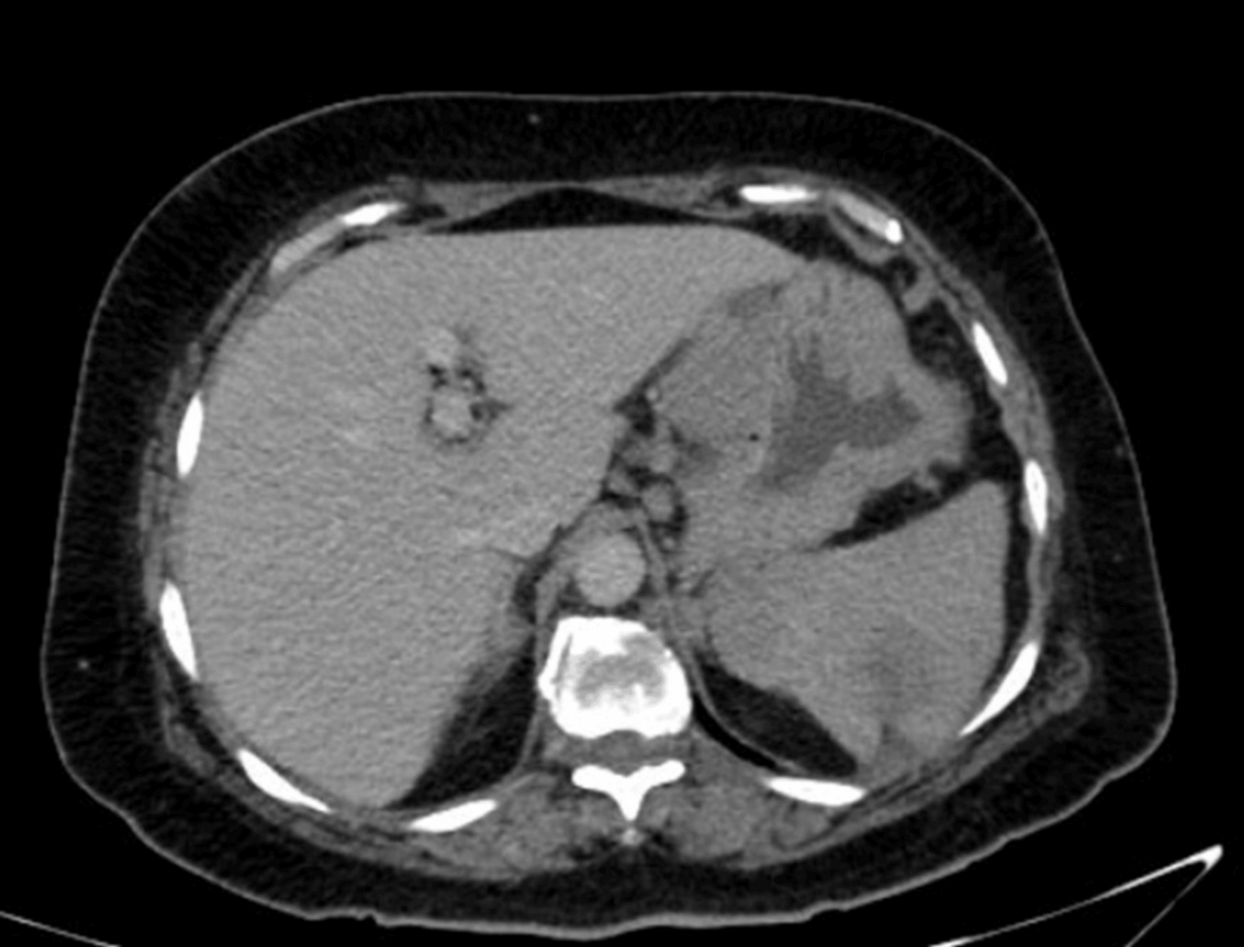
Contrast-enhanced thoracoabdominal computed tomography showing a central splenic infarct and para-aortic lymphadenopathy.

Physical examination revealed abdominal tenderness (no rigidity), fever persisted above 38.5ºC (normal range: 36.5-37ºC), consistent with HPS diagnostic criteria, and lymphadenopathy (mandibular, axillary, and inguinal). Blood, urine, and sputum cultures showed no microbial growth. Contrast-enhanced thoracoabdominal CT demonstrated splenomegaly, splenic infarcts, and lymphadenopathy (Figure 1).

During hospitalization, triglyceride levels peaked at 917 mg/dL (normal range: less than 150 mg/dL), supporting the hyperinflammatory state, accompanied by the presence of acute kidney injury with creatinine at 1.9 mg/dL (normal range: 0.59-1.04 mg/dL), blood urea nitrogen at 35 mg/dL (normal range: 6-20 mg/dL), and hyperferritinemia at 2,418.78 ng/mL (normal range: 11-307 mcg/L); LDH peaked at 3,511 U/L (normal range: 125-220 UI/L). The H-score (264 points) indicated a 99% probability of HPS.

**Figure 2:**
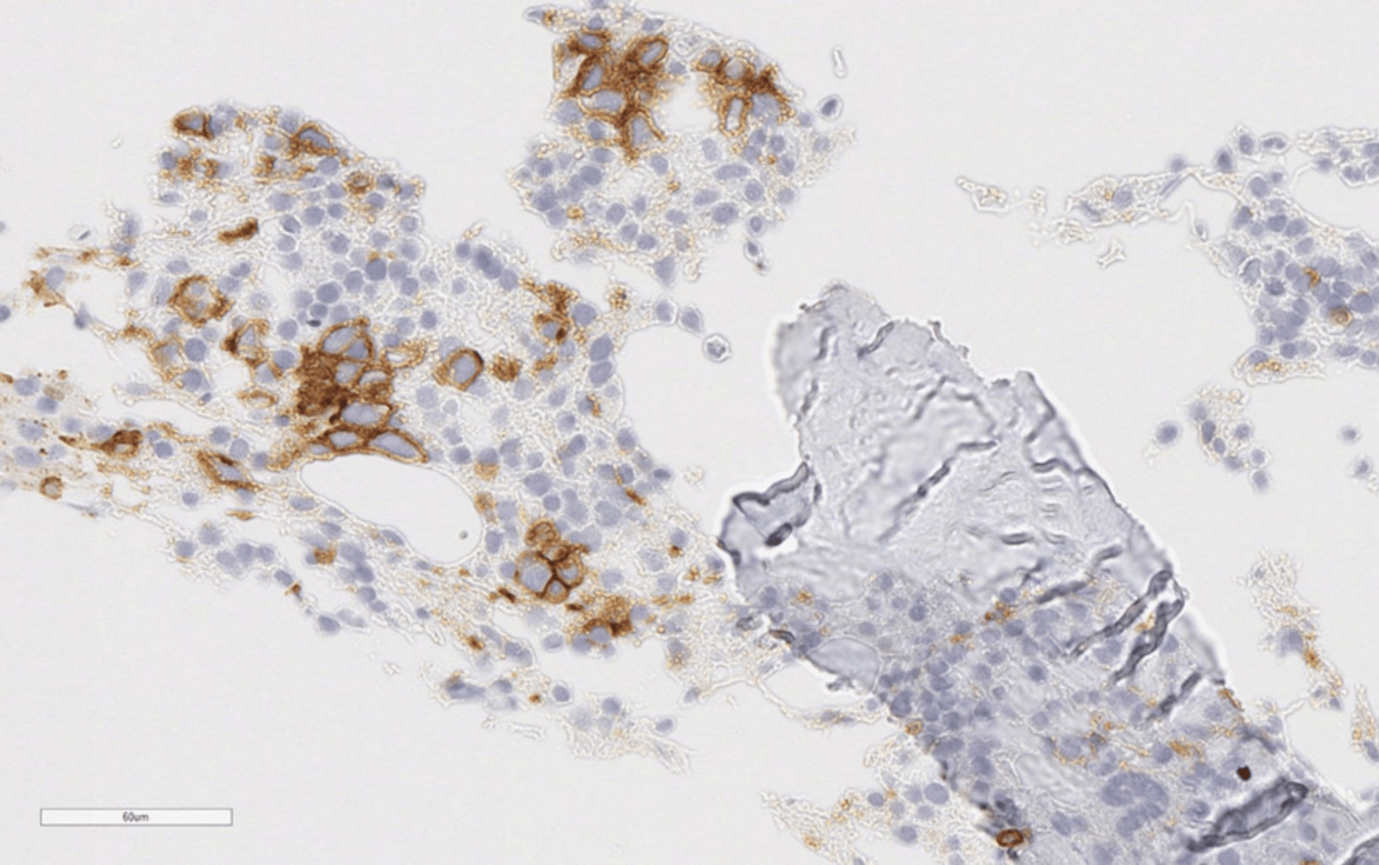
Bone marrow biopsy with immunofluorescence, showing atypical CD20-positive cells.

**Figure 3:**
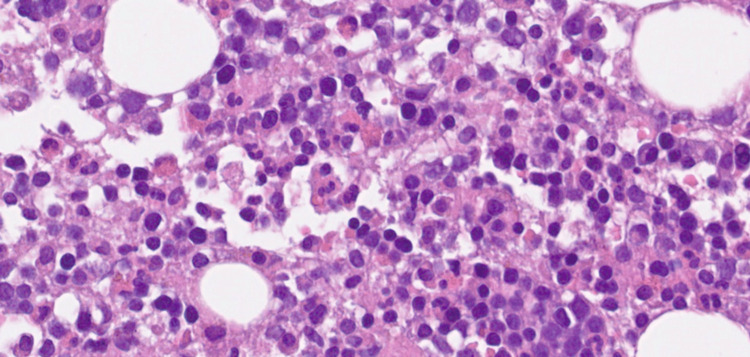
Bone marrow biopsy with hematoxylin and eosin (H&E) staining, showing hemophagocytic activity in normocellular marrow; no abnormal cells are observed in this area.

Bone marrow biopsy showed atypical CD20-positive cells (Figure 2) with hemophagocytosis (Figure 3). Axillary lymph node biopsy confirmed diffuse large B-cell lymphoma (DLBCL). 

Brain magnetic resonance imaging showed corpus callosum infiltration. The hematology department concluded this was secondary HPS caused by stage IV diffuse large B-cell non-Hodgkin lymphoma, with bone marrow and central nervous system involvement. Treatment began with a steroid, followed by doxorubicin, rituximab, cyclophosphamide, and vincristine (R-CHOP). Rapid disease progression likely contributed to the fatal outcome despite aggressive therapy.

## Discussion

The literature emphasizes the importance of prompt diagnosis and correct treatment to improve outcomes in patients with hemophagocytic syndrome. Although it is a disease more frequent in the young population, it must be considered as a differential diagnosis in critically ill patients [1, 6], especially seen as proof of poor outcomes in patients with hematologic neoplasms. Malignancy-associated HPS can appear as a primary manifestation in hematologic neoplasms. Hemophagocytic syndrome can be the result of uncontrolled tumor growth or as a consequence of a cytokine storm during cytotoxic therapy. Due to the aggressive disease courses, the initial diagnosis can be delayed [4]. Diffuse large B-cell lymphoma-associated HPS often presents with advanced-stage disease, complicating management [5].

Identifying the triggering condition is crucial to blocking the cytokine storm before eventual organ failure and death. A cutoff value of 169 for the H-score resulted in a 93% diagnostic sensitivity and an 86% specificity for HLH in adults [7].

Secondary hemophagocytic syndrome was diagnosed in our patient, triggered by non-Hodgkin lymphoma, a malignant hematologic process that can cause this syndrome in over 28.57% of patients [8]. Diagnosis was based on persistent fever, cytopenia characterized by remarkable anemia, hypertriglyceridemia with no identifiable cause, as well as hypofibrinogenemia and increased levels of ferritin [9-11]. Due to this wide range of diagnostic criteria, differential diagnosis can be hard to make; diagnoses such as infectious diseases should be considered. Lymphoma-associated HPS implies an aggressive disease course with poor prognosis, with a median survival of 11 months, even if specific therapy is applied [4]. Despite rapid diagnosis and treatment, mortality remains high (26% to 74.8%) in malignancy-associated HPS, often requiring glucocorticoids and cytotoxic therapy; it has been observed along malignancies case reports that early administration of glucocorticoids can reduce neoplasm activity, resulting in diminished tumor growth while cytotoxic therapy is started; early initiation of etoposide may improve survival in severe cases, though evidence is limited [12].

## Conclusions

Hemophagocytic syndrome predicts poor outcomes despite adequate treatment. It should be considered a key differential diagnosis in critically ill patients with worsening clinical status, even though it is uncommon in adults. Persistent inflammation unresponsive to treatment warrants its inclusion in diagnostic workups.

Hemophagocytic syndrome may serve as a prognostic marker in patients with non-Hodgkin lymphoma; its presence often signals underlying malignancy progression, necessitating urgent evaluation.
